# P-2038. Less Is More: A Targeted Blood Culture Protocol Reduces Harm and Cost in Pediatric Febrile Neutropenia

**DOI:** 10.1093/ofid/ofaf695.2202

**Published:** 2026-01-11

**Authors:** Muayad Alali

**Affiliations:** Indiana university, Carmel, Indiana

## Abstract

**Background:**

Limited evidence guides the use of repeat blood cultures beyond 48 hours in pediatric febrile neutropenia (FN), contributing to inconsistent practices. To address this gap, we implemented a protocol at Riley Hospital for Children in January 2023, limiting repeat cultures to cases of hemodynamic instability or recurrent fever (≥72 hours after defervescence).

**Figure:**
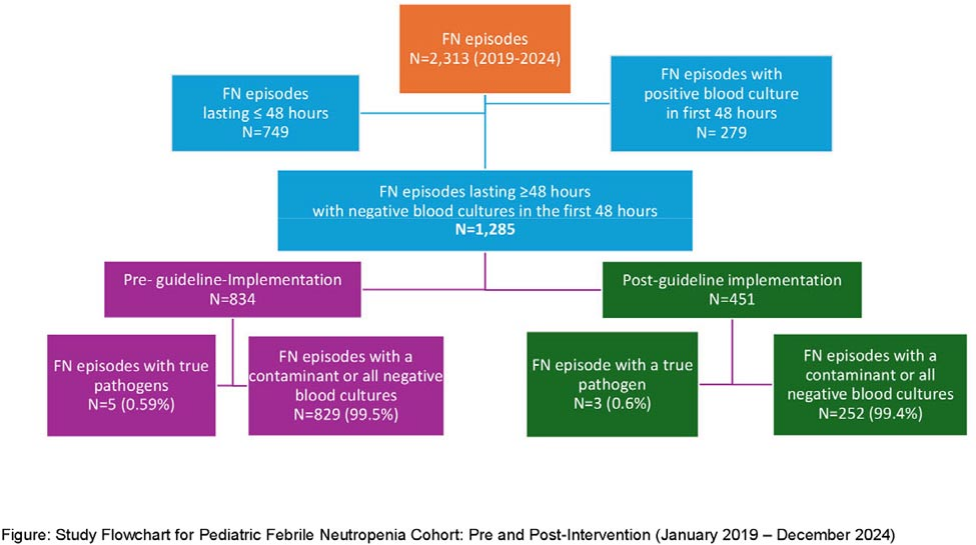

**Figure:**
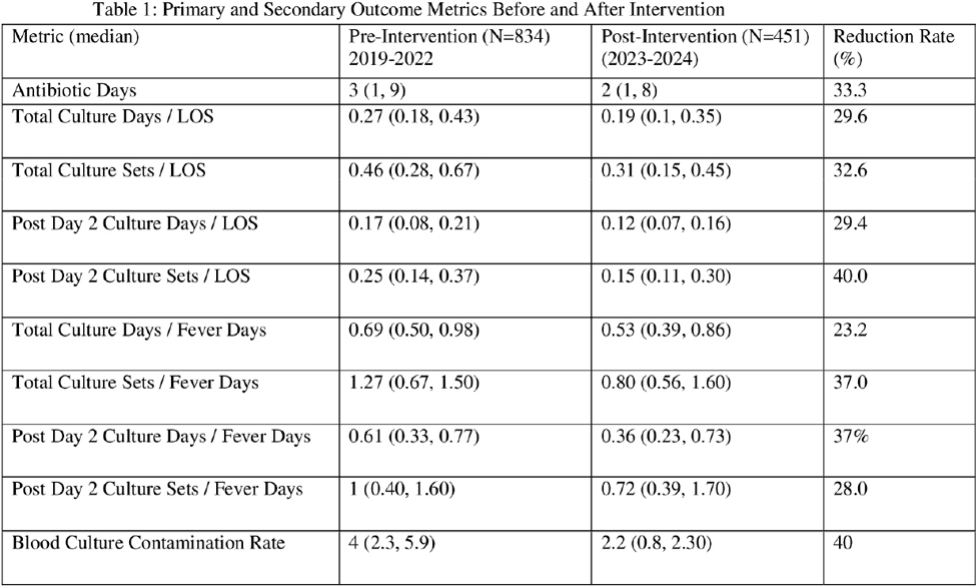

**Methods:**

Limited evidence guides the use of repeat blood cultures beyond 48 hours in pediatric febrile neutropenia (FN), contributing to inconsistent practices. To address this gap, we implemented a protocol at Riley Hospital for Children in January 2023, limiting repeat cultures to cases of hemodynamic instability or recurrent fever (≥72 hours after defervescence).

**Results:**

Of 2,313 FN episodes screened, 1,285 met inclusion criteria (834 pre- and 451 post-implementation). Among episodes with negative cultures in the first 48 hours, only 8 (0.62%) yielded new true pathogens on repeat cultures beyond 48 hours (5 pre- and 3 post-intervention). ( see Figure1). Multivariate analysis showed statistically significant reductions in antibiotic days (33%), post-Day 2 culture days per length of stay (29%), post-Day 2 culture sets per fever day (37%), and blood culture contamination rate (40%) (see Table 1) . Estimated cost savings over the two-year post-intervention period were approximately $1.5 million, primarily from reduced culture use and hospital stay. Additional savings likely stemmed from decreased antibiotic exposure, fewer contaminant workups, reduced transfusion needs, fewer missed parental workdays, and lower procedural burden and bed occupancy.

**Conclusion:**

A restrictive blood culture protocol for persistent FN was safe, low yield, and highly cost-effective, offering particular value during resource-limited periods such as the 2024 blood culture bottle shortage.

**Disclosures:**

All Authors: No reported disclosures

